# Study of cytochemical markers ACP and ANAE in childhood lymphoma and leukaemia.

**DOI:** 10.1038/bjc.1980.151

**Published:** 1980-05

**Authors:** G. Basso, M. G. Cocito, A. Poletti, C. Messina, P. Colleselli, L. Zanesco


*Br. J. Cancer* (1980) **41**, 835

***Short Communication***

### STUDY OF CYTOCHEMICAL MARKERS ACP AND ANAE IN CHILDHOOD LYMPHOMA AND LEUKAEMIA

G. BASSO*, M. G. COCITO*, A. POLETTI†, C. MESSINA*, P. COLLESELLI* AND L. ZANESCO*

*From the *Centro Leucemie Infantili—Clinica Pediatrica and †Cattedra di Oncologia, Università di Padova, Italy*

Received 8 October 1979 Accepted 3 January 1980

IMMUNOLOGICAL TECHNIQUES have shown that the lymphoproliferative malignancies of childhood are a heterogeneous group arising from lymphoid tissue. Cytochemical and immunological markers have been used to identify the various components (Brouet *et al.*, 1976; Greaves *et al.*, 1977; Brouet & Seligmann, 1978; Thiel *et al.*, 1978). Acid phosphatase (ACP) and α-naphthyl acetate acid esterase (ANAE) have revealed the difference between acute and chronic T lymphoproliferative disease and non-T malignancies (Catovsky *et al.*, 1975; Brouet *et al.*, 1975, 1976; Greaves *et al.*, 1977; Catovsky *et al.*, 1978; Thiel *et al.*, 1978; Semenzato *et al.*, 1979). In this study two cytochemical markers were used in acute lymphatic leukaemia (ALL) and in non-Hodgkin lymphoma (NHL) to assess their significance in the classification of lymphatic diseases. The simultaneous application of both enzymes for identification of different T subpopulations was also attempted.

Sixty-seven patients with acute lymphatic leukaemia (ALL) and 9 patients with non-Hodgkin lymphoma (NHL) were included in this study. Most of the patients were untreated individuals from the Centro Leucemie Infantili of the University of Padua entering clinical trials.

Immunological tests for ALL were carried out on heparinized marrow blood diluted 1:20 with RPMI 1640. For lymphomas, cell suspensions of grossly involved lymph nodes were prepared by the method of Pangalis *et al.* (1978). Aliquots of cell suspensions were used for immunological investigations, the remainder for slides with a cytospin of 200 rev/min for 15 min for cytochemical testing.

The ALL and NHL lymphoblasts were tested for spontaneous (E) rosette formation, $C_3$ receptor (EAC), and surface immunoglobulins by a direct immunofluorescence technique according to methods previously described (Semenzato *et al.*, 1979).

Acute non-lymphatic leukaemias were excluded by peroxidase (Graham & Karnovsky, 1966), non-specific esterase (Shmalzl & Braunsteiner, 1968) and chloro-esterase reactions (Moloney *et al.*, 1960). Acid phosphatase (ACP) was detected by the Schaefer *et al.* method (1975). The modified Mueller *et al.* (1975) technique was used to identify α-naphthyl acetate acid esterase (ANAE) (Semenzato *et al.*, 1979). Briefly: the smears were incubated for 3 h at 37°C in the following working solution: 2 ml hexazotized pararosaniline (prepared by mixing 50 mg pararosaniline HCl, dissolved in 3 ml 1N HCl, with 0·5 ml 1M sodium nitrite) diluted in 80 ml phosphate buffer (pH 6·55). 10 mg α-naphthyl acetate in 0·5 ml acetone was added to the buffer.

Acid phosphatase (ACP) positivity was considered when more than 70% of cells of a given patient showed an intense paranuclear reaction by criteria of Catovsky *et al.* (1975, 1978). ANAE$^+$ cells were assessed on the presence of cytoplasmic granules. More than 30% of cells with brown cytoplasmic granules in ALL and lymphoma cultures were considered $ANAE^+$ by the Kulenkampff *et al.* (1977) grading of postnatal thymocytes.

The ALL and NHL were divided into 4 groups according to the results of immunological investigations.

The T group included 10 cases of ALL showing a percentage of E-rosetting cells ranging between 29 and 78% in the marrow, and 2 cases of NHL with 42 and 64% E-rosetting cells in the suspensions obtained from grossly involved lymph nodes.

The B group is composed from 3 NHL presenting with a high percentage of blasts with surface immunoglobulins: 48, 50 and 59% respectively.

The $T_{EAC+}$ group consisted of 4 patients with ALL and 2 with NHL whose blasts formed E and EAC rosettes simultaneously, in percentages varying from 65 to 85%.

The non-T non-B group included 53 ALL and 2 NHL with no immunological markers.

Acid phosphatase (ACP) was positive in all the T and $T_{EAC+}$ cell malignancies and in 3 non-T non-B ALL (Figure). The percentage of $ACP^+$ cells varied from 70 to 98%. The percentage of $ACP^+$ cells in the B and the remainder of the non-T and non-B lymphoproliferative diseases was always < 56% (Figure) and usually < 25%.

FIGURE.—Correlation between immunological and cytochemical markers; simultaneous evaluation of ACP and ANAE activity in T (×), B (○), $T_{EAC+}$ (▲) and non-T non-B (●) malignancies.

High ANAE activity (> 30%) was found in the T malignancies and in 3 cases of non-T non-B, $ACP^+$ ALL lymphatic leukaemias (Figure). Fifty-three cases of non-T non-B ALL and the 6 cases of $T_{EAC+}$ malignancies were $ANAE^-$. The percentage of $ANAE^+$ cells was almost 20%. Simultaneous $ACP^+$ and $ANAE^+$ reactions occurred at high levels in the T malignancies and in 3 ALL non-T non-B type. The $T_{EAC+}$ cases had high values for ACP only (> 95%). In the remaining 55 malignancies the two enzymes were present in small percentages (Figure).

The classification of lymphoproliferative diseases by immunological methods is of great clinical importance especially for prognostic purposes (Belpomme *et al.*, 1977; Borella *et al.*, 1977; Coccia *et al.*, 1976; Chessels *et al.*, 1977; Catovsky *et al.*, 1978). Cytochemical markers provide a valid contribution to the correct diagnosis.

Catovsky (1975) has shown that an intense ACP reaction (in the paranuclear zone) of more than 70% of cells is characteristic of T ALL. These results were confirmed later by the same author (Catovsky *et al.*, 1978) and other groups (Brouet *et al.*, 1976; Thiel *et al.*, 1978). Furthermore, a positive ACP reaction was noted in foetal thymocytes as early as the 12th week of gestation (Stein & Muller-Hermelink, 1977) and in some ALL cases with T antigens, but without E rosetting (Thiel *et al.*, 1978).

This study confirms the capacity of ACP to mark not only T cells but also early T cells that are positive in $T_{EAC+}$ syndromes as well. In fact Stein & Muller-Hermelink (1977) demonstrated the correlation between $T_{EAC+}$ lymphoblasts and human foetal thymocytes of a 12–16-week-old foetus.

Mueller *et al.* (1975) identified ANAE as a T-cell marker in the lymph nodes of rats. Positivity is high in circulating T lymphocytes (Ranki *et al.*, 1976; Knowles *et al.*, 1978; Basso *et al.*, 1980), moderate ($\sim 30\%$) in human postnatal thymocytes (Kulenkampff *et al.*, 1977; Basso *et al.*, 1980) and almost negative in foetal thymocytes before 20 weeks of gestation (Basso *et al.*, 1980). These results are confirmed in the present study. In fact, the ANAE is moderately positive in T-cell ALL and lymphomas but absent in $T_{EAC+}$ and non-T-cell malignancies.

The 3 cases of non-T non-B ALL with double positivity presented clinically as a T-cell form with mediastinal mass and raised peripheral white-cell count. We suggest that these 3 cases may be of T ALL type the blasts of which had undergone changes in their membrane during the leukaemic process with subsequent loss of sheep red blood cell receptors. This is in part confirmed by the observation that one of 10 T ALL with high rosetting and enzyme positivity at its onset lost spontaneous E-rosette formation but maintained the double enzyme activity during relapse. The importance of the combined ACP and ANAE tests is stressed since it is the only valid method available for differentiating cytochemically the T, $T_{EAC+}$, non-T non-B cell lymphoproliferative diseases. In fact, with ACP alone T and non-T tumours can be distinguished, but not a T ALL or a T lymphoma from these $T_{EAC+}$ types. Furthermore, $T_{EAC+}$ syndromes cannot be differentiated from non-receptor ones by ANAE alone. The 2 enzymes are necessary for identification of 2 T-cell subpopulations. One is positive for ACP only and the other for both. These two subpopulations could indicate various stages of T-cell differentiation. The two enzymes could also be possible markers for some T-negative ALL, as seen by the 3 ALL cases described above.

### REFERENCES

Basso, G., Cocito, M. G., Semenzato, G., Pezzutto, A. & Zanesco, L. (1980) Cytochemical study of thymocytes and T lymphocytes. *Br. J. Haematol.* (In press.)

Belpomme, D., Lelarge, N., Mathé, G. & Davies, A. J. S. (1977) Etiological, clinical and prognostic significance of the T-B immunological classification of primary acute lymphoid leukaemias and non-Hodgkin's lymphomas. In *Immunological Diagnosis of Leukaemias and Lymphomas.* Eds Thierfelder, Rodt & Thiel. Vol. 20. Berlin: Springer Verlag. p. 33.

Borella, L., Sen, L., Dow, L. W. & Casper, J. T. (1977) Cell differentiation antigens *versus* tumor-related antigens in childhood actue lymphoblastic leukaemia (ALL). Clinical significance of leukaemia markers. In *Immunological Diagnosis of Leukaemias and Lymphomas.* Eds Thierfelder, Rodt & Thiel. Vol. 20. Berlin: Springer Verlag. p. 77.

Brouet, J. C., Flandrin, G., Sasportes, M., Preud'Homme, J. L. & Seligmann, M. (1975). Chronic lymphocytic leukaemia of T-cell origin. An immunological and clinical evaluation in eleven patients. *Lancet*, ii, 890.

Brouet, J. C., Valensi, F., Daniel, M. T., Flandrin, G., Preud'Homme, J. L. & Seligmann, M. (1976) Immunological classification of acute lymphoblastic leukaemias: Evaluation of histoclinical significance in a hundred patients. *Br. J. Haematol.*, **33**, 319.

Brouet, J. C. & Seligmann, M. (1978) The immunological classification of acute lymphoblastic leukaemias. *Cancer*, **42**, 817.

Catovsky, D. (1975) T-cell origin of acid phosphatase positive lymphoblasts. *Lancet*, ii, 327.

Catovsky, D., Greaves, M. F., Pain, C., Cherchi, M., Janossy, G. & Kay, H. E. M. (1978) Acid-phosphatase reaction in acute lymphoblastic leukaemia. *Lancet*, i, 749.

Chessels, J. M., Hardisty, R. M., Rapson, N. T. & Greaves, M. E. (1977) Acute lymphoblastic leukaemia in children: Classification and prognosis. *Lancet*, ii, 1307.

Coccia, P. F., Kersey, J. H., Gajl-Peczalska, K. J., Krivit, W. & Nesbit, M. E. (1976) Prognostic significance of surface marker analyses in childhood non Hodgkin's lymphoproliferative malignancies. *Am. J. Hematol.*, **1**, 405.

Graham, R. C. & Karnovsky, M. J. (1966) The early stages of injected horse-radish peroxidase in the proximal tubules of mouse kidney ultrastructural cytochemistry by a new technique. *J. Histochem. Cytochem.*, **67**, 291.

Greaves, M. F., Janossy, G., Roberts, M. & 5 others (1977) Membrane phenotyping: diagnosis, monitoring and classification of acute "lymphoid" leukaemias. In *Immunological Diagnosis of Leukaemias and Lymphomas.* Eds Thierfelder, Rodt & Thiel. Vol. 20. Berlin: Springer Verlag. p. 61.

Knowles, D. M., Hoffman, N. T., Ferrarini, M. & Kunkel, H. G. (1978) The demonstration of acid α-naphthyl acetate esterase activity in human lymphocytes: Usefulness as a T-cell marker. *Cell. Immunol.*, **35**, 112.

Kulenkampff, J., Janossy, G. & Greaves, M. F. (1977) Acid esterase in human lymphoid cells and leukemic blasts: a marker for T lymphocytes. *Br. J. Haematol.*, **36**, 231.

Moloney, W. C., McPherson, K. & Fliegelman, L. (1960) Esterase activity in leucocytes demonstrated by the use of naphthol AS-D chloroacetate substrate. *J. Histochem. Cytochem.*, **8**, 200.

MUELLER, J., BRUN, DEL RE, G., BUERKI, H., KELLER, U. G., HESS, M. W. & COTTIER, H. (1975) Non specific acid esterase activity: a criterion of T and B lymphocytes in mouse lymphnodes. *Eur. J. Immunol.*, **5**, 270.

PANGALIS, G A., WALDMAN, S. R. & RAPPAPORT, H. (1978) Cytochemical findings in human non neoplastic blood and tonsillar B and T lymphocytes. *Am. J. Clin. Pathol.*, **69**, 314.

RANKI, A., TÖTTERMAN, T. H. & HÄVRY, P. (1976) Identification of resting human T and B lymphocytes by acid α-naphthyl acetate esterase staining combined with rosette formation with *Staphylococcus aureus* strain Cowan 1. *Scand. J. Immunol.*, **5**, 1129.

SCHAEFER, H. E., HELLRIEGEL, K. P., ZACH, J. & FISCHER, R. (1975) Zytochemischer polymorphismus der sauren phosphatase be haarzell-leukamie. *Blut*, **31**, 365.

SCHMALZL, F. & BRAUNSTEINER, H. (1968) On the origin of monocytes. *Acta Haematol.*, **39**, 177.

SEMENZATO, G., AMADORI, G., BASSO, G. & 4 others (1979) Immunological features in chronic lymphocytic leukaemia (CLL) of T cell origin. *J. Clin. Lab. Immunol.*, **2**, 45.

STEIN, H. & MULLER-HERMELINK, H. K. (1977) Simultaneous presence of receptors for complement and sheep blood cells on human foetal thymocytes. *Br. J. Haematol.*, **36**, 225.

THIEL, E., RODT, H., NETZEL, B. & 5 others (1978) T-zell-antigen positive, E-rosetten negative akute lymphoblasten leukämie. *Blut*, **36**, 363.


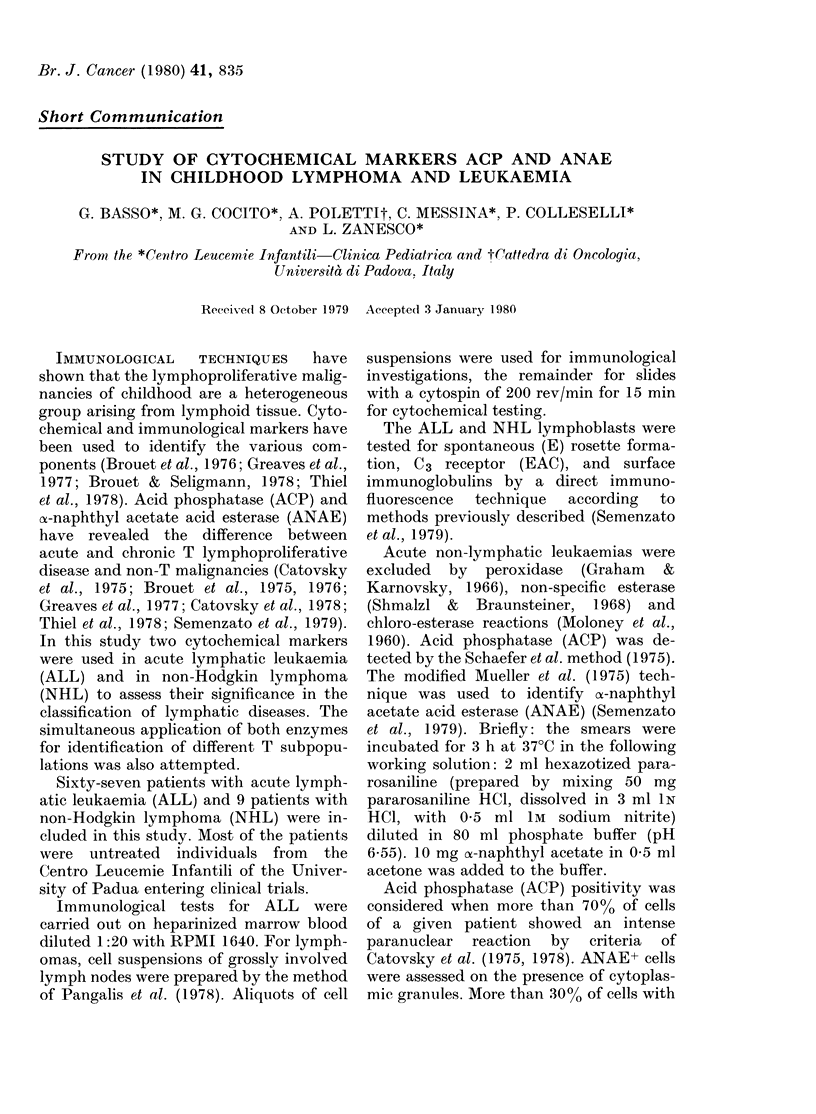

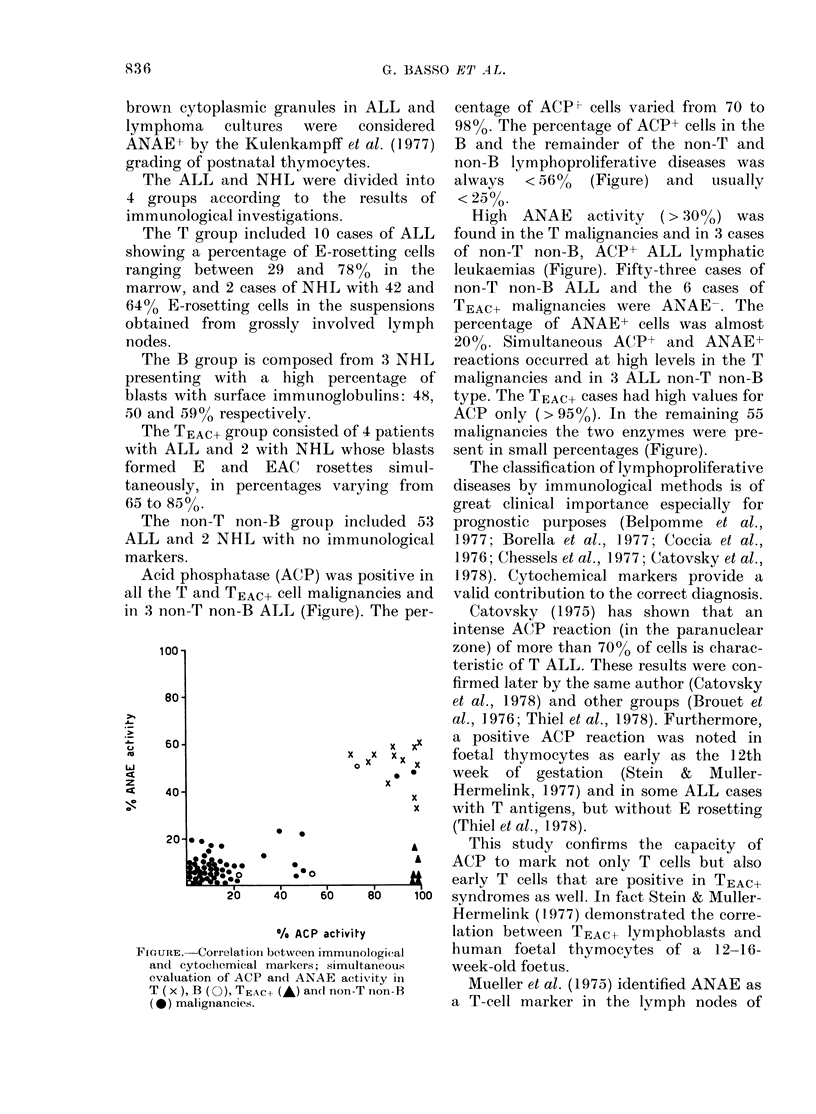

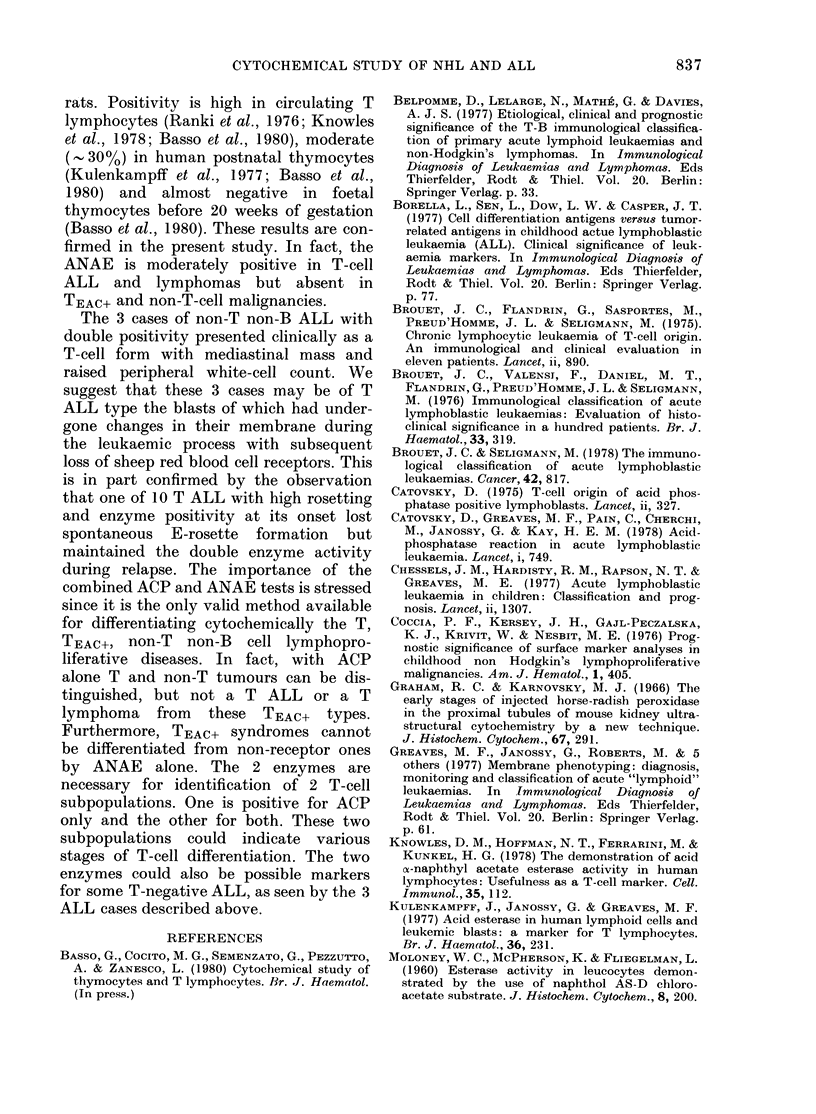

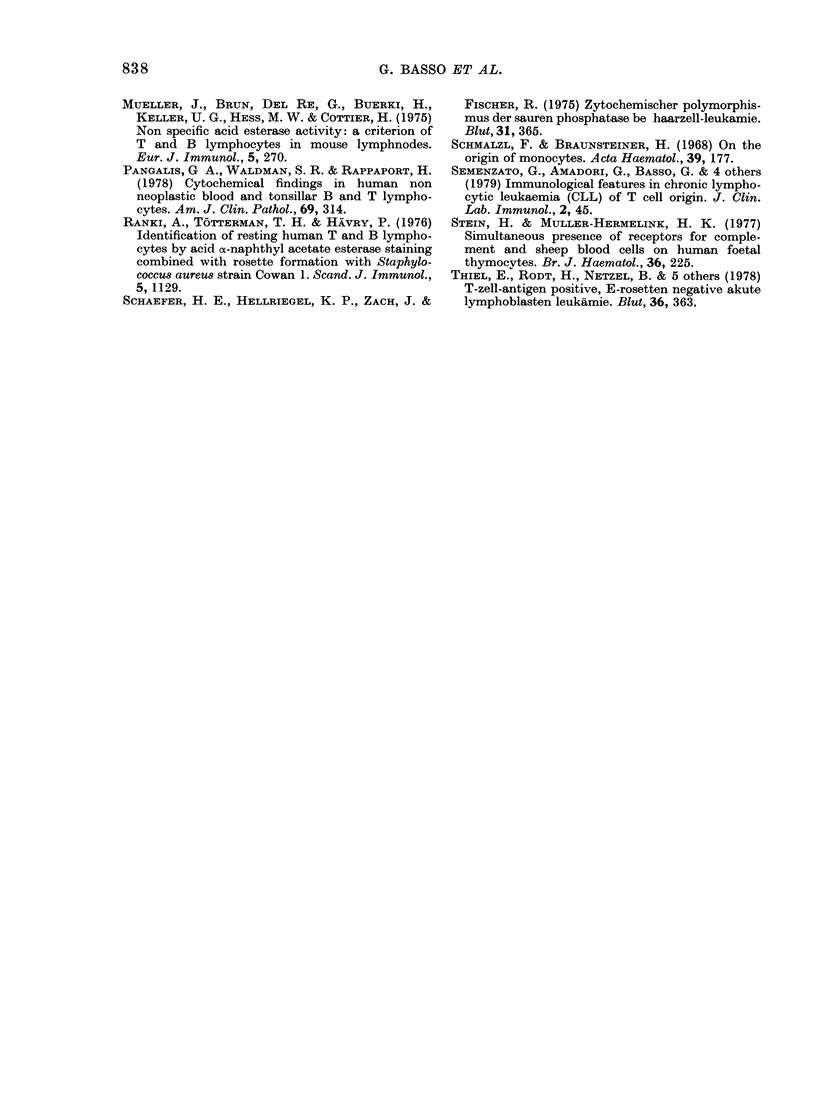


## References

[OCR_00313] Brouet J. C., Sasportes M., Flandrin G., Preud'Homme J. L., Seligmann M. (1975). Chronic lymphocytic leukaemia of T-cell origin. Immunological and clinical evaluation in eleven patients.. Lancet.

[OCR_00328] Brouet J. C., Seligmann M. (1978). The immunological classification of acute lymphoblastic leukemias.. Cancer.

[OCR_00320] Brouet J. C., Valensi F., Daniel M. T., Flandrin G., Preud'homme J. L., Seligmann M. (1976). Immunological classification of acute lymphoblastic leukaemias: evaluation of its clinical significance in a hundred patients.. Br J Haematol.

[OCR_00337] Catovsky D., Cherchi M., Greaves M. F., Janossy G., Pain C., Kay H. E. (1978). Acid-phosphatase reaction in acute lymphoblastic leukaemia.. Lancet.

[OCR_00333] Catovsky D. (1975). Letter: T-cell origin of acid-phosphatase-positive lymphoblasts.. Lancet.

[OCR_00343] Chessells J. M., Hardisty R. M., Rapson N. T., Greaves M. F. (1977). Acute lymphoblastic leukaemia in children: Classification and prognosis.. Lancet.

[OCR_00349] Coccia P. F., Kersey J. H., Gajil-Peczalska K. J., Krivit W., Nesbit M. E. (1976). Prognostic significance of surface marker analysis in childhood non-Hodgkin's lymphoproliferative malifnancies.. Am J Hematol.

[OCR_00356] Graham R. C., Karnovsky M. J. (1966). The early stages of absorption of injected horseradish peroxidase in the proximal tubules of mouse kidney: ultrastructural cytochemistry by a new technique.. J Histochem Cytochem.

[OCR_00372] Knowles D. M., Hoffman T., Ferrarini M., Kunkel H. G. (1978). The demonstration of acid alpha-naphthyl acetate esterase activity in human lymphocytes: usefulness as a T-cell marker.. Cell Immunol.

[OCR_00379] Kulenkampff J., Janossy G., Greaves M. F. (1977). Acid esterase in human lymphoid cells and leukaemic blasts: a marker for T lymphocytes.. Br J Haematol.

[OCR_00385] MOLONEY W. C., MCPHERSON K., FLIEGELMAN L. (1960). Esterase activity in leukocytes demonstrated by the use of naphthol AS-D chloroacetate substrate.. J Histochem Cytochem.

[OCR_00395] Mueller J., Brun del Re G., Buerki H., Keller H. U., Hess M. W., Cottier H. (1975). Nonspecific acid esterase activity: a criterion for differentiation of T and B lymphocytes in mouse lymph nodes.. Eur J Immunol.

[OCR_00400] Pangalis G. A., Waldman S. R., Rappaport H. (1978). Cytochemical findings in human nonneoplastic blood and tonsillar B and T lymphocytes.. Am J Clin Pathol.

[OCR_00406] Ranki A., Tötterman T. H., Häyry P. (1976). Identification of resting human T and B lymphocytes by acid alpha-naphthyl acetate esterase staining combined with rosette formation with Staphylococcus aureus strain Cowan 1.. Scand J Immunol.

[OCR_00414] Schaefer H. E., Hellriegel K. P., Zach J., Fischer R. (1975). Zytochemischer Polymorphismus der sauren Phosphatase bei Haarzell-Leukämie. Blut.

[OCR_00420] Schmalzl F., Braunsteiner H. (1968). [On the origin of monocytes].. Acta Haematol.

[OCR_00430] Stein H., Müller-Hermelink H. K. (1977). Simultaneous presence of receptors for complement and sheep red blood cells on human fetal thymocytes.. Br J Haematol.

[OCR_00436] Thiel E., Rodt H., Netzel D., Huhn D., Wündisch G. F., Hass R. J., Bender-Götze C., Thierfelder S. (1978). T-Zell-Antigen positive, E-Rosetten negative akute Lymphoblastenleukämie.. Blut.

